# Supplementary material for: Source of Dietary Fat in Pig Diet Affects Adipose Expression of Genes Related to Cancer, Cardiovascular, and Neurodegenerative Diseases
Source: Genes (Basel). 2019 Nov 20;10(12):948. doi: 10.3390/genes10120948 (PMC6947373; doi:10.3390/genes10120948)
Supplement: Supplementary file 1 [file genes-10-00948-s001.pdf]

Supplementary table 1. Functional enrichments identified among DEGs from rapeseed oil vs beef tallow comparison with DAVID software

Category	Term	Count	P value	Genes
KEGG_PATHWAY	hsa04610: Complement and coagulation cascade	3	0.004	THBD,F3,PLAU
REACTOME_PATHWAY	R-HSA-1236973: Crosspresentation of particulate exogenous antigens (phagosomes) (Homo sapiens)	2	0.013	CYBB,NCF2
REACTOME_PATHWAY	R-HSA-5668599: RHO GTPases Activate NADPH Oxidases (Homo sapiens)	2	0.021	CYBB,NCF2
REACTOME_PATHWAY	R-HSA-1222556: ROS, RNS production in phagocytes (Homo sapiens)	2	0.055	CYBB,NCF2
REACTOME_PATHWAY	R-HSA-4420097: VEGFAVEGFR2 Pathway (Homo sapiens)	2	0.098	CYBB,NCF2
GOTERM_BP_DIRECT	GO:0002479~antigen processing and presentation of exogenous peptide and presentation of exogenous peptide antigen via MHC class I, TAP-dependent	3	0.004	CYBB,NCF2,PSME4
GOTERM_BP_DIRECT	GO:0045730~respiratory burst	2	0.019	CYBB,NCF2
GOTERM_BP_DIRECT	GO:0042554~superoxide anion generation	2	0.021	CYBB,NCF2
GOTERM_BP_DIRECT	GO:0006801~superoxide metabolic process		0.029	CYBB,NCF2
GOTERM_BP_DIRECT	GO:0007596~blood coagulation	3	0.030	THBD,F3,PLAU
GOTERM_BP_DIRECT	GO:0022008~neurogenesis	2	0.061	ZNF217,CHAC1
GOTERM_BP_DIRECT	GO:0045732~positive regulation of protein catabolic processes	2	0.086	PLK2,TIPARP
	GO:0032092~positive			

GOTERM_BP_DIRECT	regulation of protein binding	2	0.087	PLK2,PLXND1
GOTERM_CC_DIRECT	GO:0043020~NADPH oxidase complex	2	0.018	CYBB,NCF2
GOTERM_CC_DIRECT	GO:0009986~cell surface	4	0.045	THBD,SRPX2,F3,PLAU
GOTERM_MF_DIRECT	GO:0016175~superoxide-generating NADPH oxidase activity	2	0.017	CYBB,NCF2
GOTERM_MF_DIRECT	GO:0005515~protein binding	20	0.042	NCF2,CHAC1,MAFB, FKBP5,FST,CTCFL, TSC22D1,USP53,CYBB, 2ZNF217,RAB18, PLK2,SRPX2,F3,PSME4, PLXND1,PLAU, SPP1,CD200R1

Supplementary Figure 1. MA Plots obtained after analysis of RNA-seq with DESeq 2 software.

Rapeseed oil vs Beef tallow

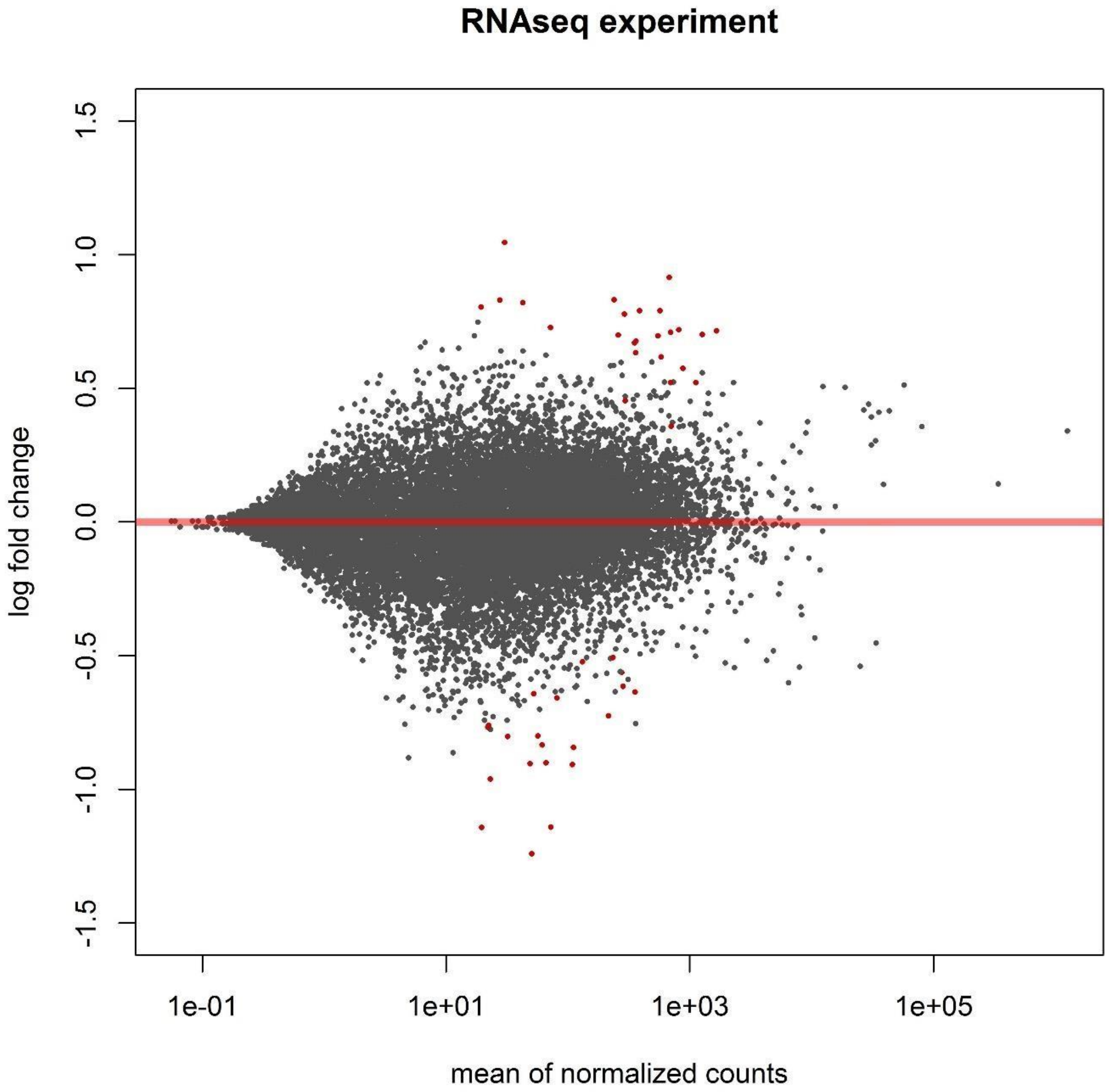

Rapeseed oil vs Coconut oil

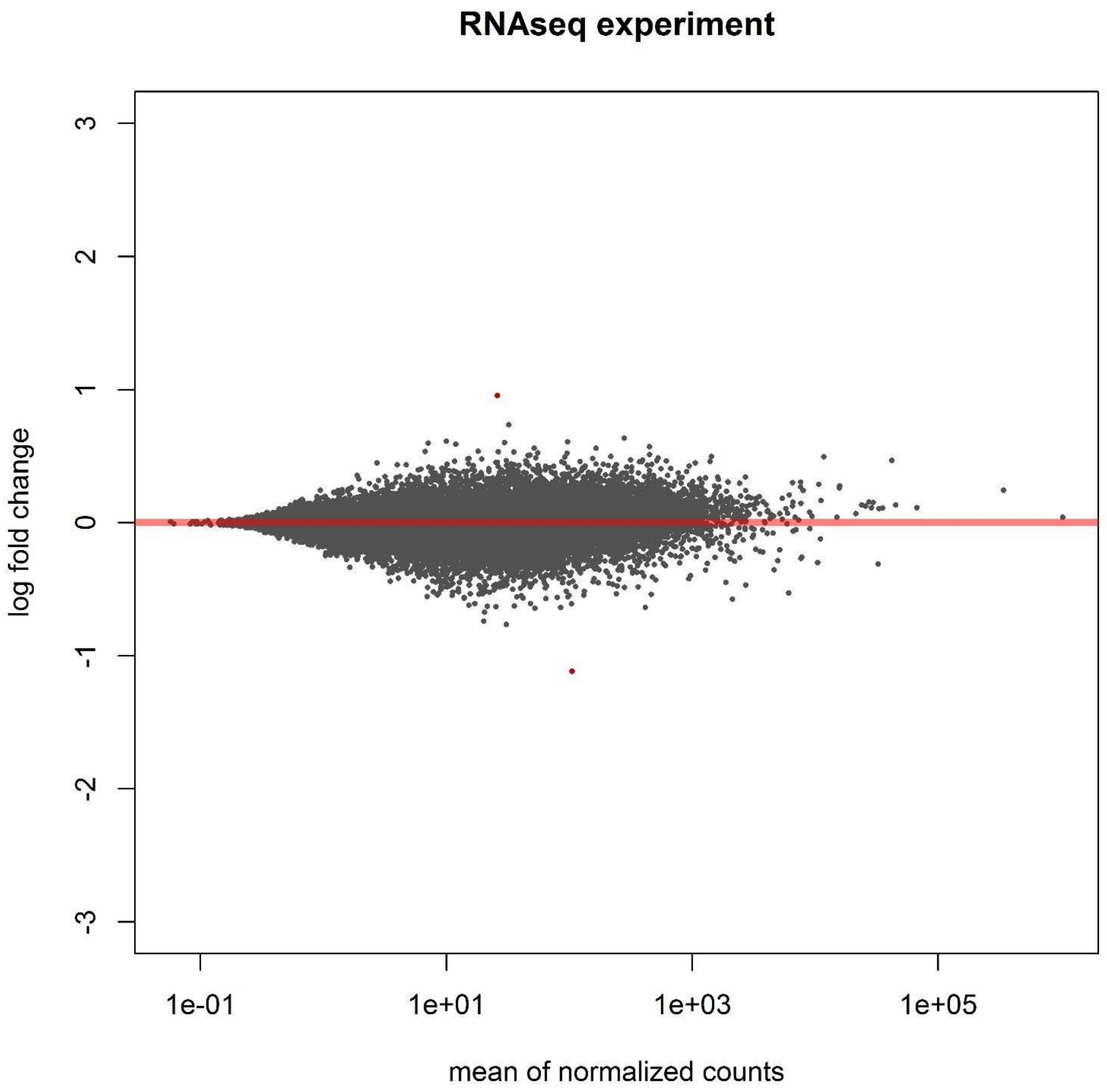

RNAseq experiment
log fold change
3
2
1
0
-1
-2
-3
1e-01
1e+01
1e+03
1e+05
mean of normalized counts

Beef tallow vs Coconut oil

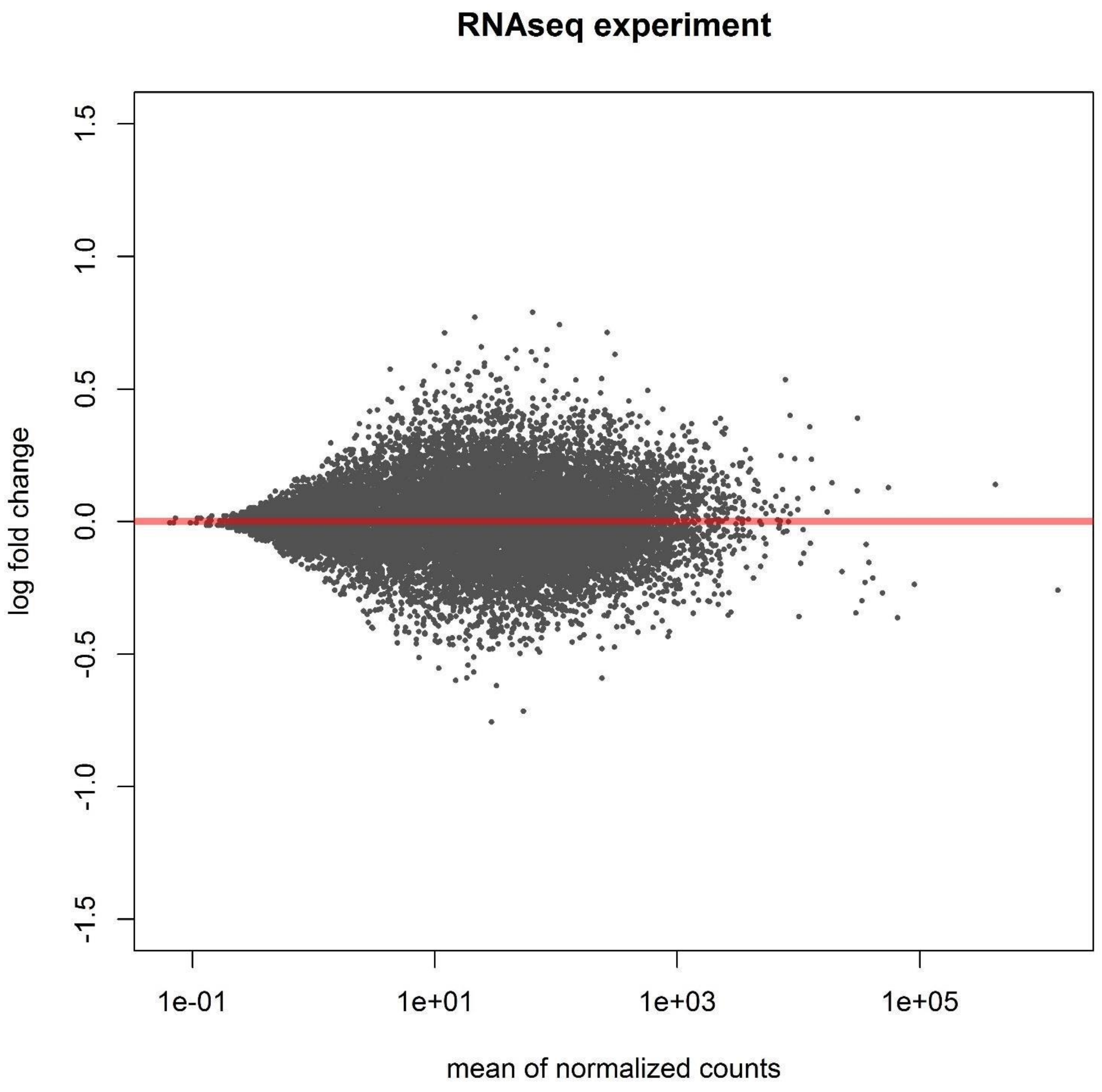

Supplementary Figure 2. PCA plot showing sample relationship based on RNA-seq data.

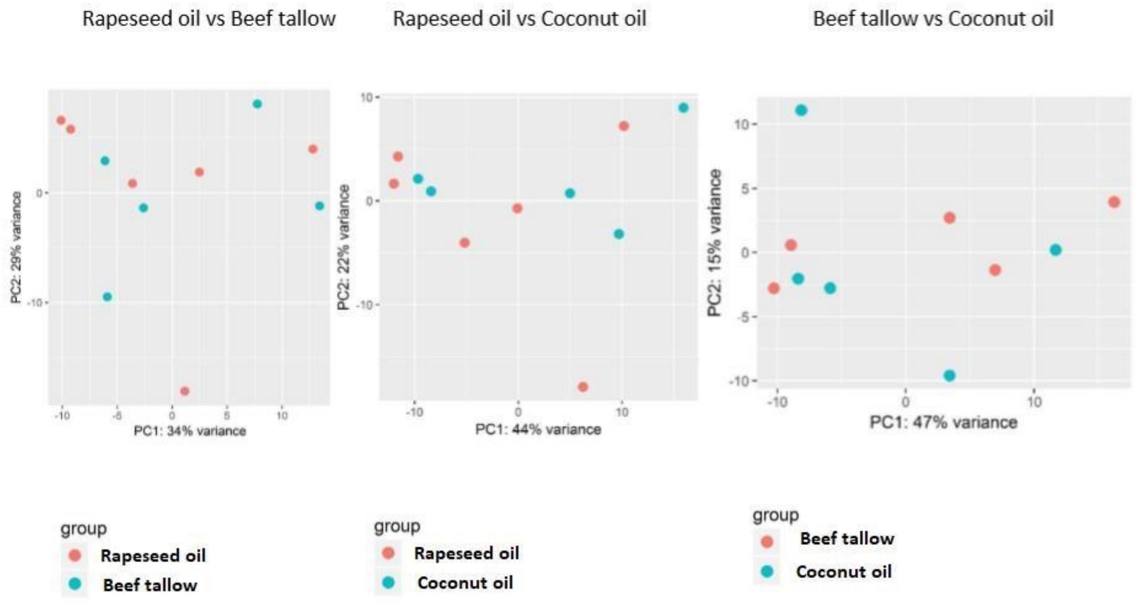

Supplementary figure 3. Graphical presentation of results of the GSEA obtained with WebGestalt software

Rapeseed oil vs Beef tallow

Biological Processes

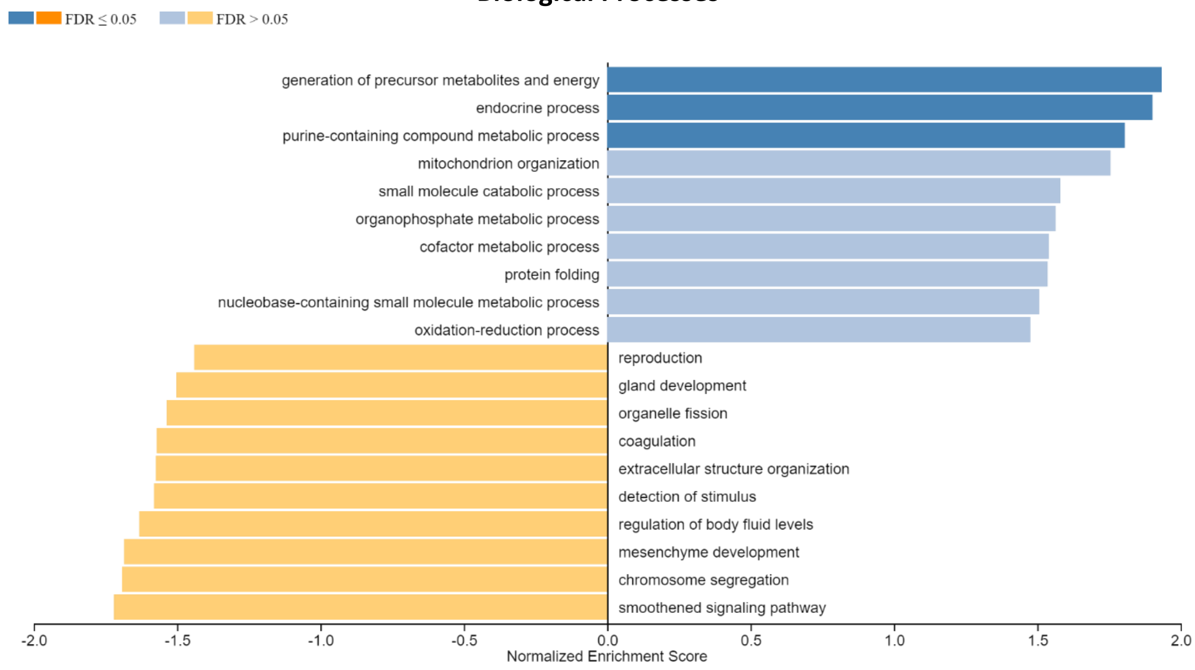

Rapeseed oil vs Beef tallow

Cellular components

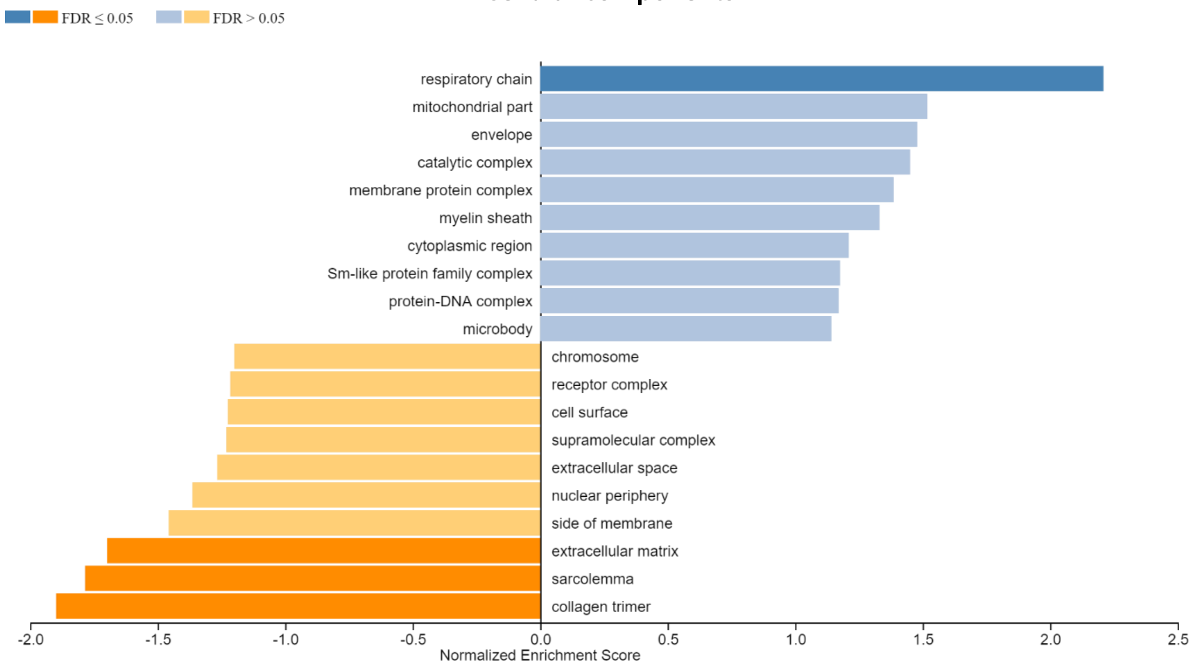

Rapeseed oil vs Beef tallow

Molecular function

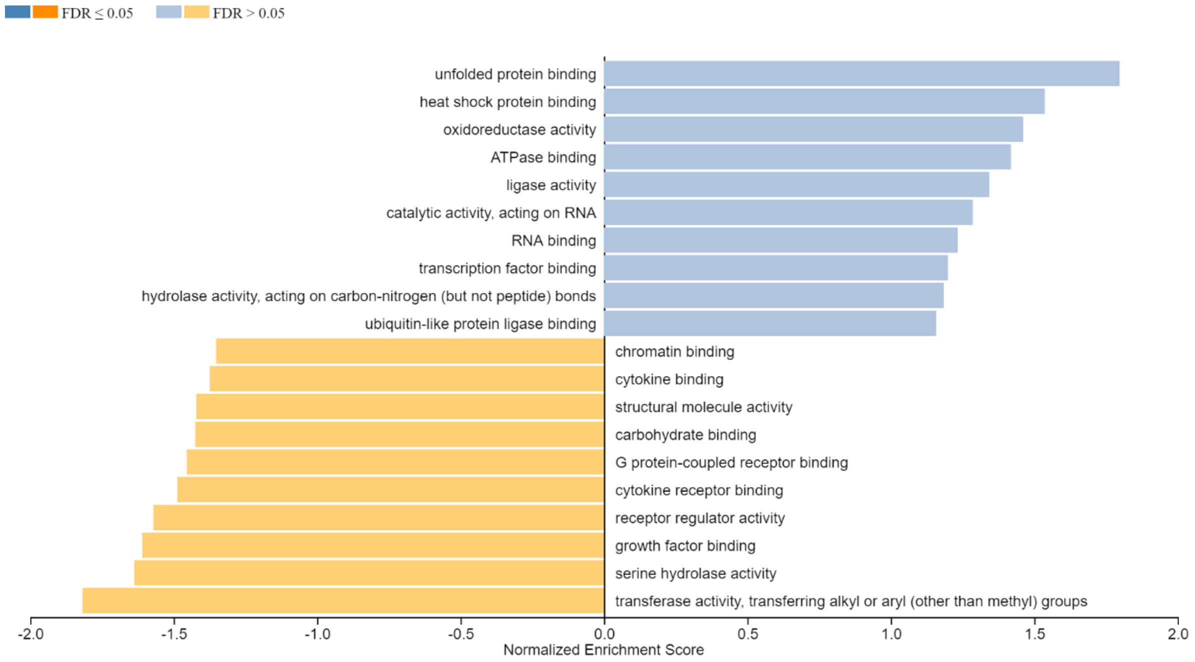

Rapeseed oil vs Coconut oil KEGG pathways

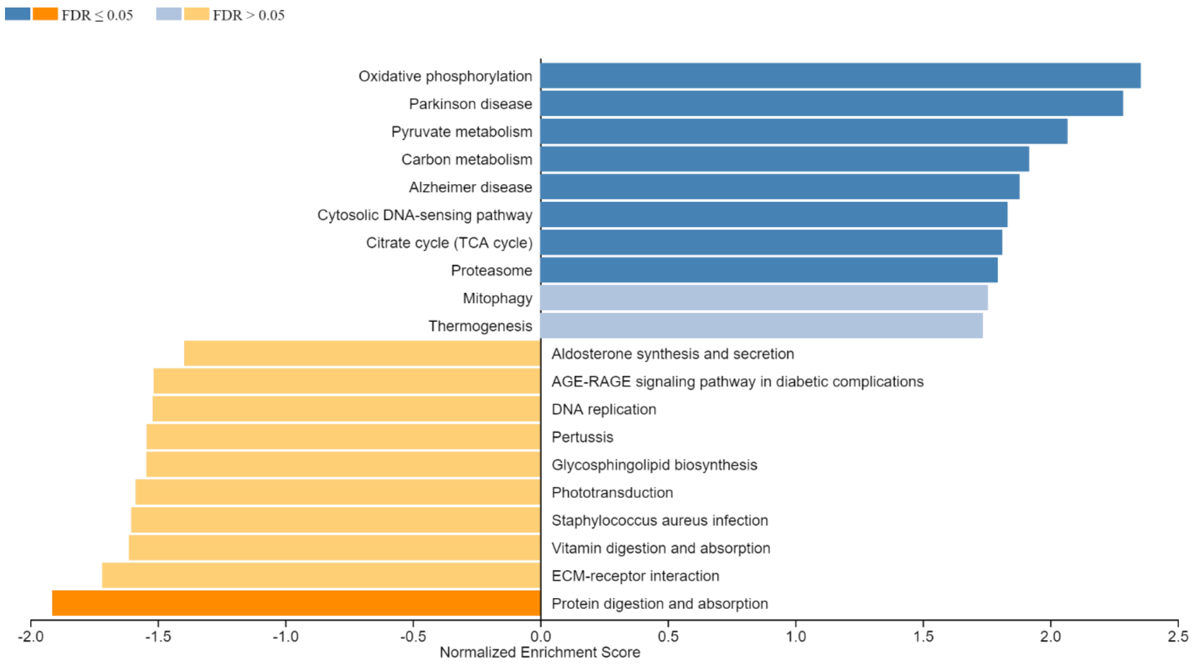

Rapeseed oil vs Coconut oil

Wiki pathways

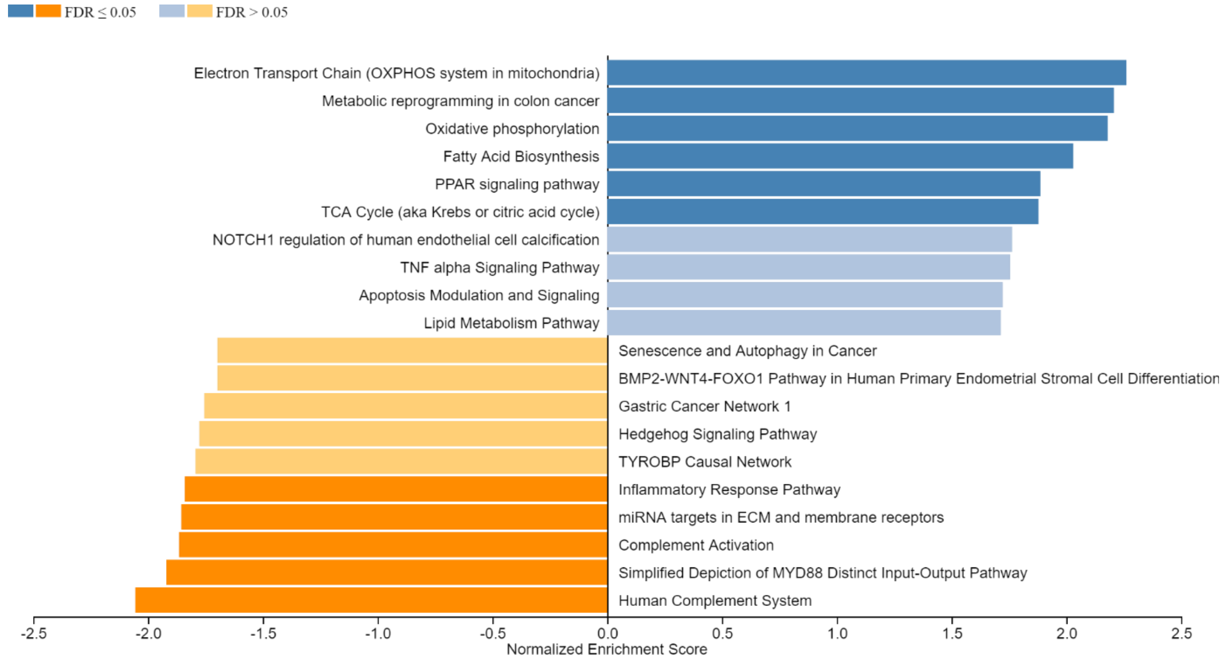

Rapeseed oil vs Coconut oil

Biological processes

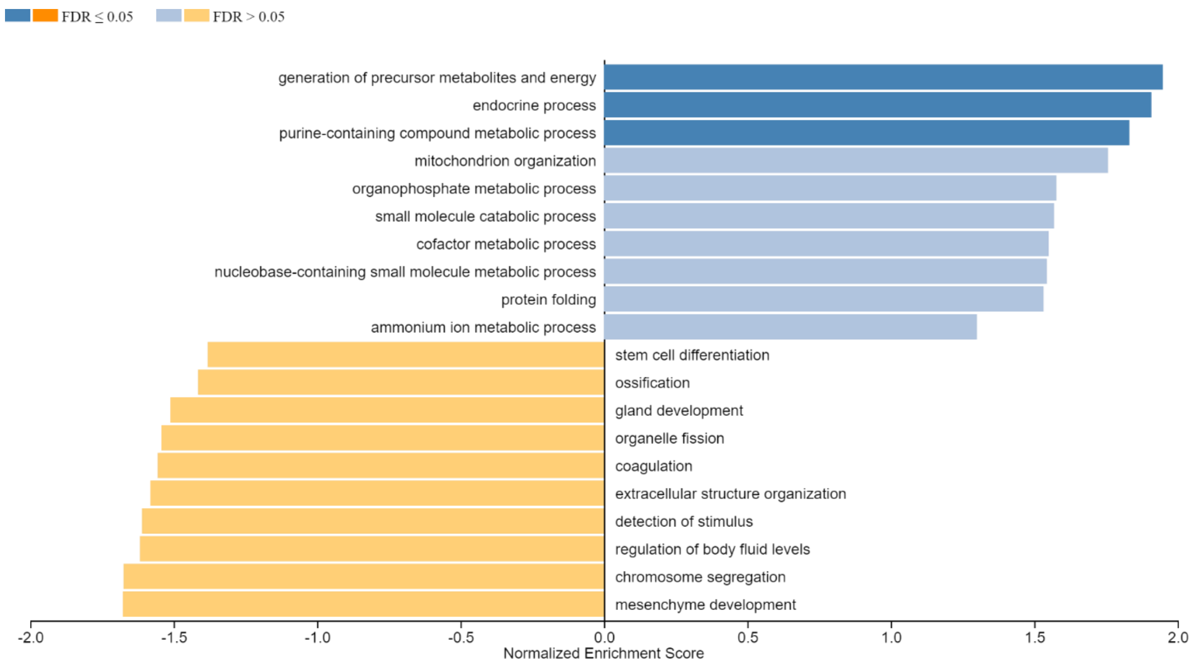

Rapeseed oil vs Coconut oil

Cellular component

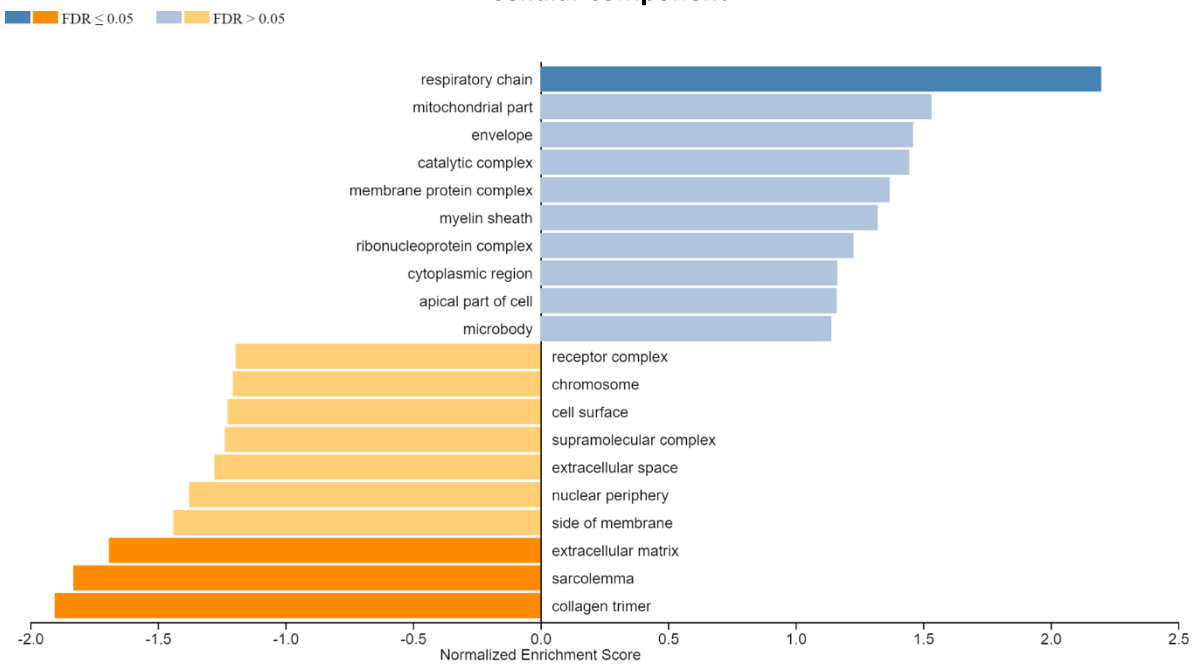

Rapeseed oil vs Coconut oil

Molecular function

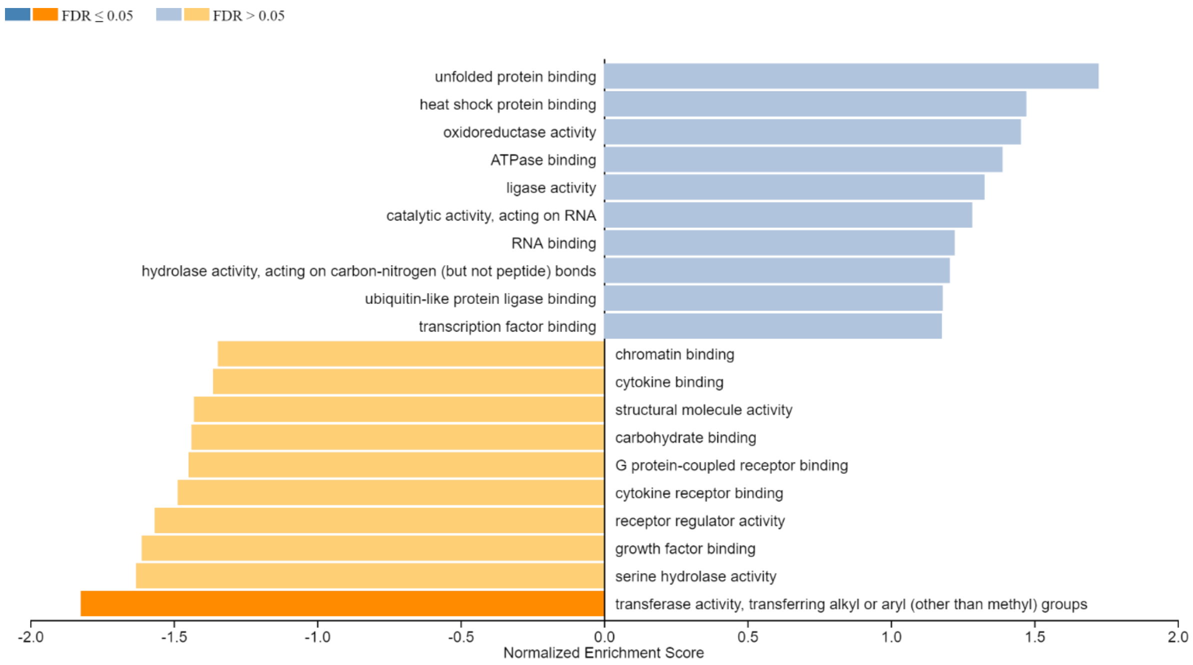

Beef tallow vs Coconut oil KEGG pathways

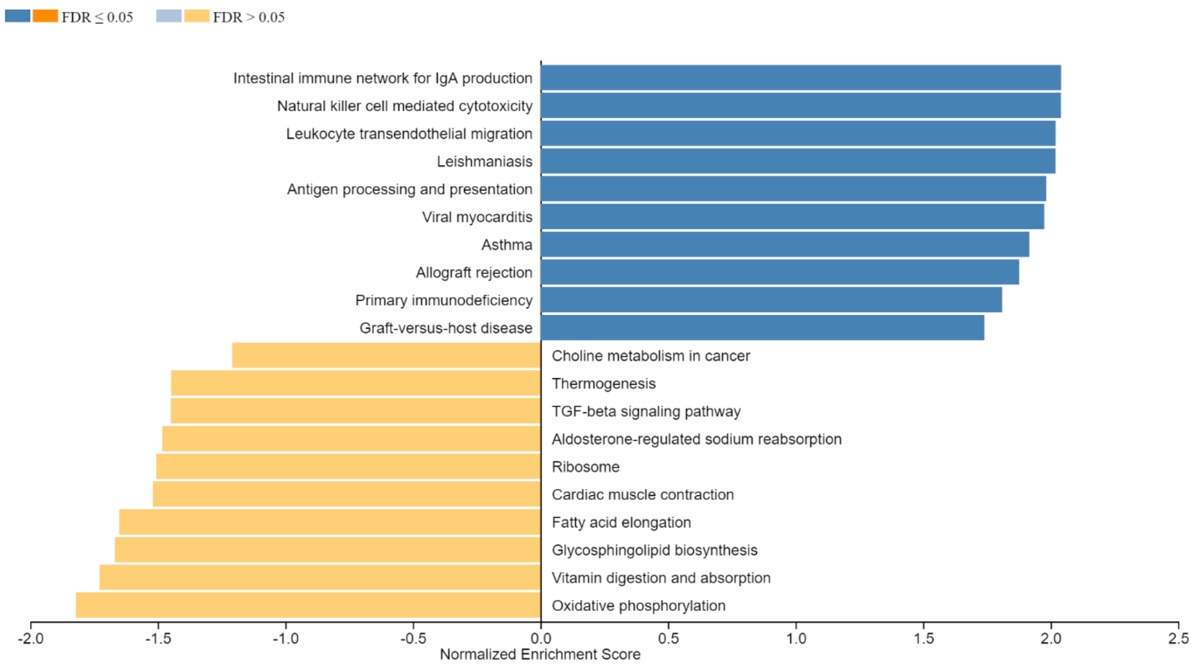

Beef tallow vs Coconut oil

Wikipathways (Homo sapiens)

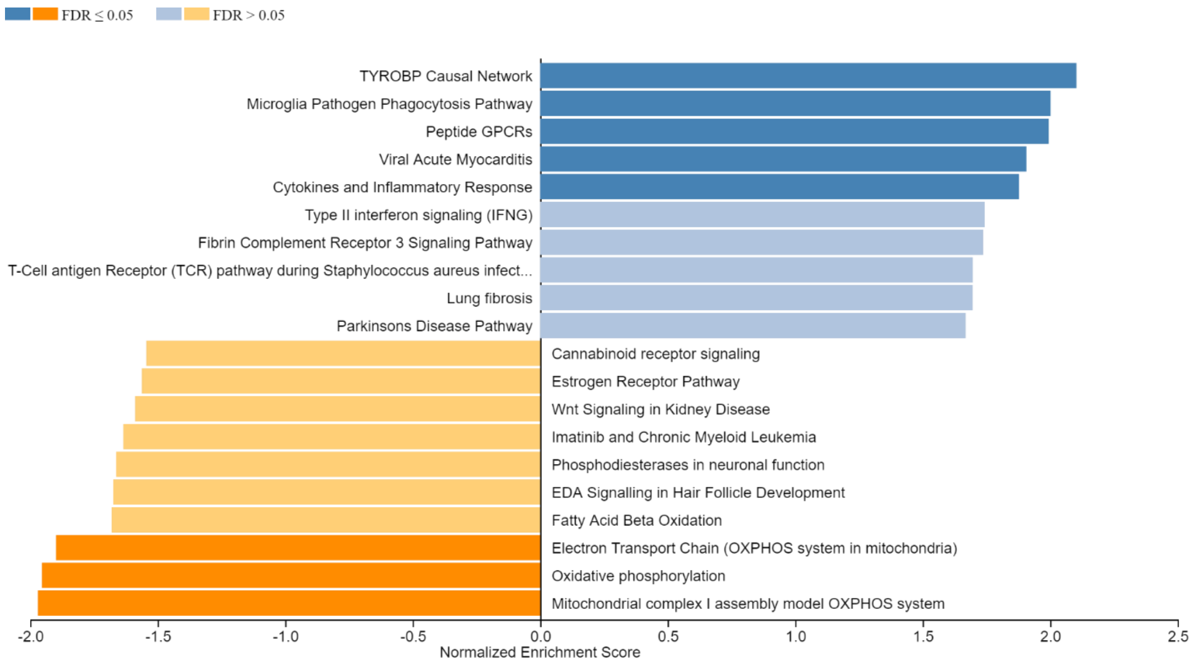

Beef tallow vs Coconut oil

Biological processes

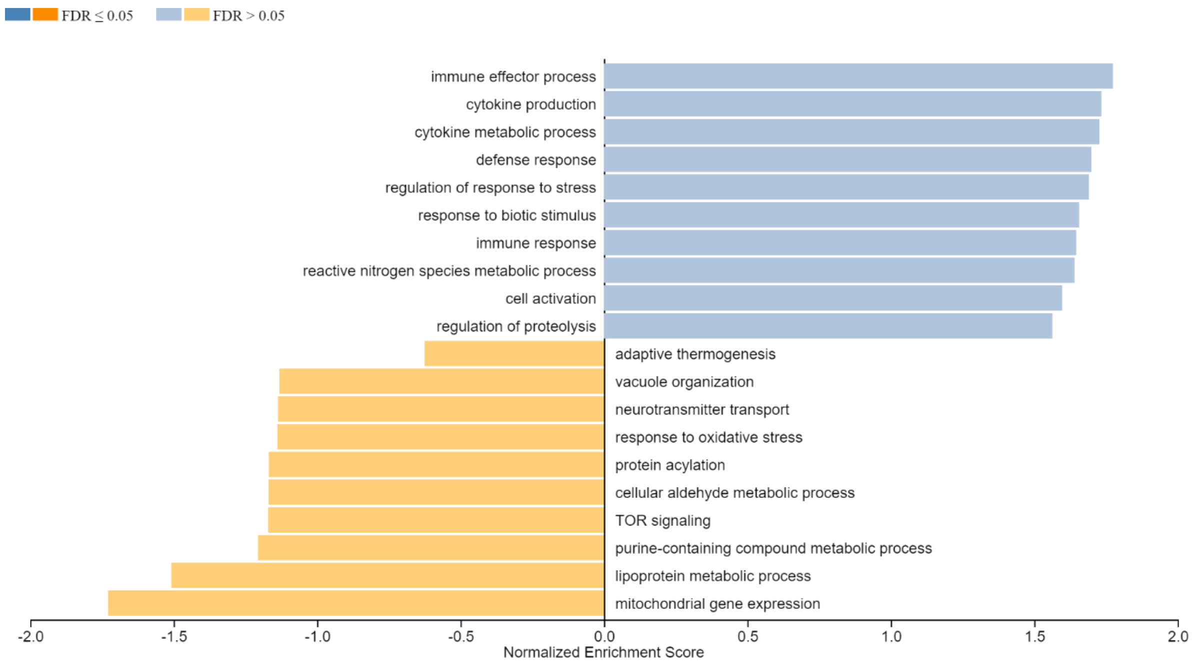

Beef tallow vs Coconut oil

Cellular component

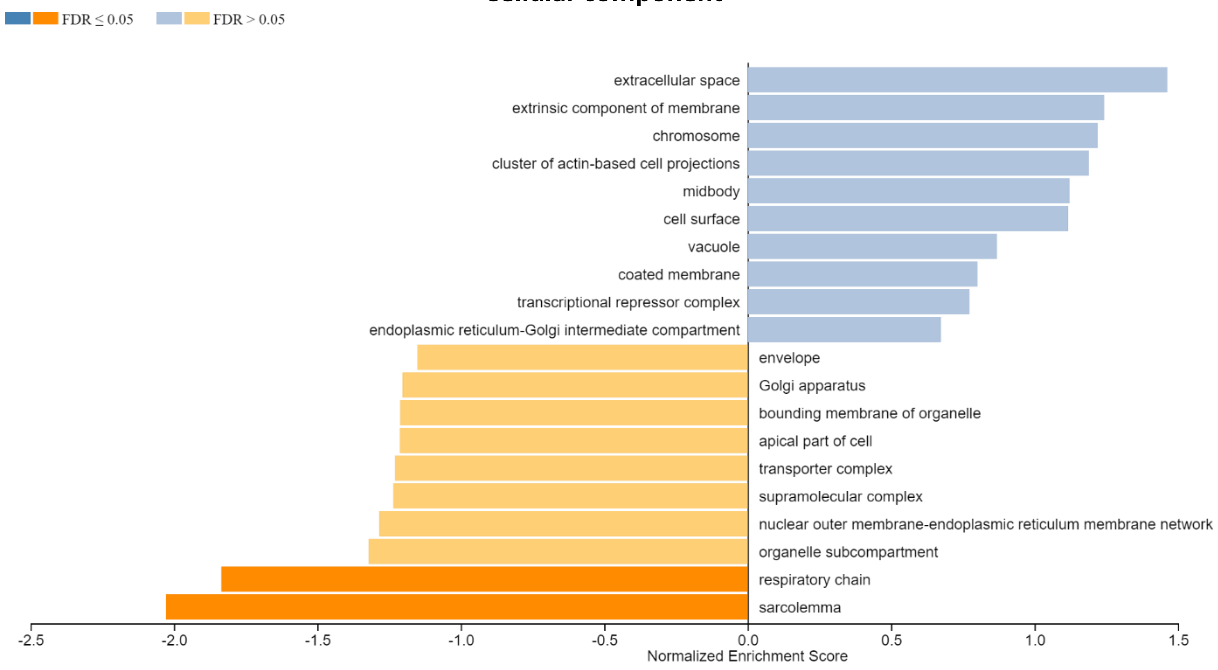

Beef tallow vs Coconut oil Molecular function

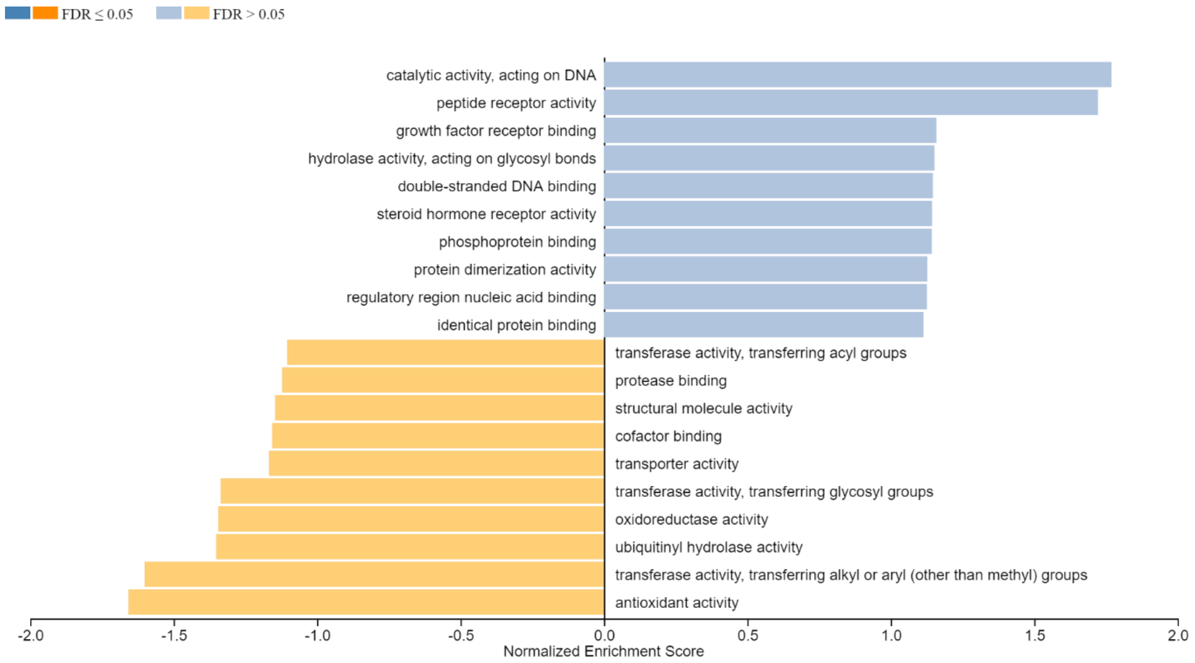

Supplementary figure 4. Scatter plot of RNA-seq vs qPCR results.

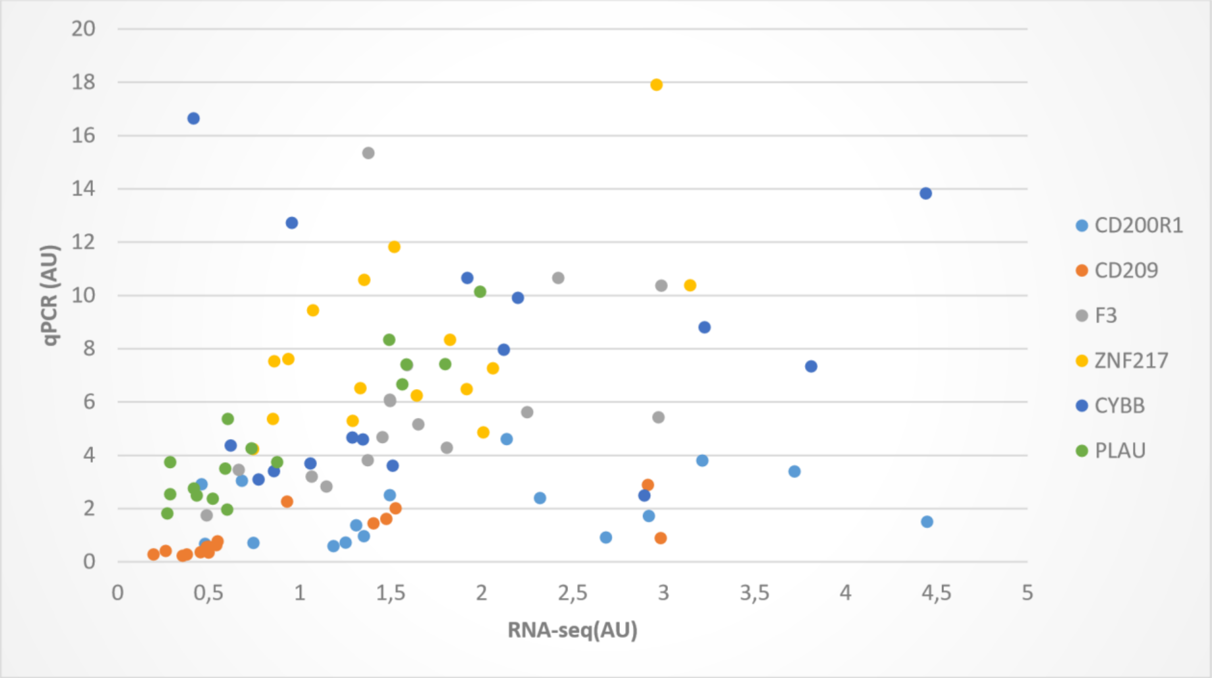

Supplementary Table 2. Results of the GSEA obtained with Web Gestalt software. ES-Enrichment score, NES- Normalized Enrichment Score

**R_vs_B_KEGG**					
geneSet	description	ES	NES	pValue	FDR
ssc00190	Oxidative phosphorylation	0.71	2.75	0.0000	0.0000
ssc03050	Proteasome	0.64	1.91	0.0023	0.0107
ssc05012	Parkinson disease	0.58	2.30	0.0000	0.0000
ssc05010	Alzheimer disease	0.57	2.27	0.0000	0.0000
ssc00071	Fatty acid degradation	0.55	1.81	0.0024	0.0290
ssc04723	Retrograde endocannabinoid signaling	0.50	2.03	0.0000	0.0028
ssc04920	Adipocytokine signaling pathway	0.50	1.79	0.0000	0.0299
ssc04932	Non-alcoholic fatty liver disease (NAFLD)	0.50	1.98	0.0000	0.0042
ssc04714	Thermogenesis	0.49	2.12	0.0000	0.0003
ssc05016	Huntington disease	0.45	1.85	0.0000	0.0192
ssc04145	Phagosome	-0.50	-1.86	0.0000	0.0143
ssc05321	Inflammatory bowel disease (IBD)	-0.56	-1.80	0.0033	0.0203
ssc05140	Leishmaniasis	-0.60	-1.94	0.0000	0.0048
ssc05416	Viral myocarditis	-0.60	-1.78	0.0033	0.0191
ssc04650	Natural killer cell mediated cytotoxicity	-0.61	-2.06	0.0000	0.0018
ssc03030	DNA replication	-0.66	-1.84	0.0000	0.0149
ssc05150	Staphylococcus aureus infection	-0.70	-2.04	0.0000	0.0018

ssc04672	Intestinal immune network for IgA production	-0.74	-1.96	0.0000	0.0051
ssc05330	Allograft rejection	-0.74	-1.86	0.0018	0.0165
ssc05332	Graft-versus-host disease	-0.79	-1.79	0.0037	0.0203
**R_vs_B_WIKI**					
geneSet	description	ES	NES	pValue	FDR
WP3413	NOTCH1 regulation of human endothelial cell calcification	0.80	2.09	0.0000	0.0018
WP623	Oxidative phosphorylation	0.73	2.42	0.0000	0.0000
WP143	Fatty Acid Beta Oxidation	0.70	2.05	0.0000	0.0024
WP4324	Mitochondrial complex I assembly model OXPHOS system	0.70	2.35	0.0000	0.0000
WP111	Electron Transport Chain (OXPHOS system in mitochondria)	0.68	2.60	0.0000	0.0000
WP2453	TCA Cycle and Deficiency of Pyruvate Dehydrogenase complex (PDHc)	0.68	1.73	0.0069	0.0863
WP357	Fatty Acid Biosynthesis	0.64	1.82	0.0023	0.0453
WP4290	Metabolic reprogramming in colon cancer	0.59	2.01	0.0000	0.0041
WP500	Glycogen Metabolism	0.52	1.73	0.0025	0.0917
WP4396	Nonalcoholic fatty liver disease	0.47	1.92	0.0000	0.0117
WP2328	Allograft Rejection	-0.54	-1.78	0.0000	0.0337
WP45	G1 to S cell cycle control	-0.57	-1.81	0.0049	0.0332
WP2806	Human Complement System	-0.57	-1.99	0.0000	0.0046
WP2446	Retinoblastoma Gene in Cancer	-0.62	-2.13	0.0000	0.0015
WP3937	Microglia Pathogen Phagocytosis Pathway	-0.65	-1.93	0.0000	0.0107
WP466	DNA Replication	-0.66	-1.96	0.0000	0.0069
WP2361	Gastric Cancer Network 1	-0.67	-1.85	0.0000	0.0257

WP3945	TYROBP Causal Network	-0.71	-2.28	0.0000	0.0000
WP530	Cytokines and Inflammatory Response	-0.74	-1.88	0.0000	0.0193
WP3287	Overview of nanoparticle effects	-0.79	-1.84	0.0037	0.0259
**R_vs_B_BP**					
geneSet	description	ES	NES	pValue	FDR
GO:0050886	endocrine process	0.67	1.90	0.0023	0.0209
GO:0006091	generation of precursor metabolites and energy	0.47	1.93	0.0000	0.0238
GO:0006457	protein folding	0.43	1.54	0.0215	0.2296
GO:0072521	purine-containing compound metabolic process	0.43	1.81	0.0000	0.0485
GO:0007005	mitochondrion organization	0.43	1.75	0.0000	0.0623
GO:0044282	small molecule catabolic process	0.39	1.58	0.0056	0.2534
GO:0051186	cofactor metabolic process	0.36	1.54	0.0060	0.2530
GO:0055086	nucleobase-containing small molecule metabolic process	0.34	1.51	0.0068	0.2518
GO:0019637	organophosphate metabolic process	0.33	1.56	0.0000	0.2431
GO:0055114	oxidation-reduction process	0.31	1.48	0.0038	0.2903
GO:0000003	reproduction	-0.32	-1.44	0.0000	0.4736
GO:0048732	gland development	-0.38	-1.50	0.0093	0.3215
GO:0048285	organelle fission	-0.40	-1.54	0.0064	0.2691
GO:0050878	regulation of body fluid levels	-0.44	-1.63	0.0048	0.2082
GO:0043062	extracellular structure organization	-0.44	-1.57	0.0096	0.2496
GO:0007059	chromosome segregation	-0.45	-1.69	0.0016	0.2256
GO:0060485	mesenchyme development	-0.46	-1.68	0.0031	0.1629

GO:0050817	coagulation	-0.46	-1.57	0.0105	0.2205
GO:0051606	detection of stimulus	-0.53	-1.58	0.0125	0.2820
GO:0007224	smoothened signaling pathway	-0.56	-1.72	0.0134	0.3244
**R_vs_B_CC**					
geneSet	description	ES	NES	pValue	FDR
GO:0070469	respiratory chain	0.67	2.21	0.0000	0.0000
GO:0032993	protein-DNA complex	0.37	1.17	0.2049	0.2925
GO:0120114	Sm-like protein family complex	0.36	1.18	0.2076	0.3107
GO:0043209	myelin sheath	0.36	1.33	0.0791	0.1972
GO:0042579	microbody	0.36	1.14	0.2602	0.3159
GO:0044429	mitochondrial part	0.33	1.52	0.0037	0.1664
GO:0031975	envelope	0.32	1.48	0.0000	0.1494
GO:0099568	cytoplasmic region	0.30	1.21	0.1216	0.3169
GO:1902494	catalytic complex	0.29	1.45	0.0000	0.1359
GO:0098796	membrane protein complex	0.29	1.39	0.0000	0.1655
GO:0005694	chromosome	-0.29	-1.20	0.1010	0.5863
GO:0005615	extracellular space	-0.29	-1.27	0.0432	0.6209
GO:0009986	cell surface	-0.30	-1.22	0.0892	0.6174
GO:0099080	supramolecular complex	-0.30	-1.23	0.0994	0.6772
GO:0043235	receptor complex	-0.33	-1.22	0.1329	0.5883
GO:0098552	side of membrane	-0.37	-1.46	0.0089	0.2212
GO:0034399	nuclear periphery	-0.45	-1.36	0.0819	0.3708

GO:0031012	extracellular matrix	-0.46	-1.70	0.0000	0.0381
GO:0042383	sarcolemma	-0.63	-1.78	0.0068	0.0199
GO:0005581	collagen trimer	-0.76	-1.90	0.0000	0.0115
**R_vs_B_MF**					
geneSet	description	ES	NES	pValue	FDR
GO:0051082	unfolded protein binding	0.54	1.80	0.0000	0.0765
GO:0031072	heat shock protein binding	0.50	1.54	0.0165	0.3281
GO:0051117	ATPase binding	0.49	1.42	0.0686	0.3714
GO:0016874	ligase activity	0.37	1.34	0.0781	0.4791
GO:0016810	hydrolase activity. acting on carbon-nitrogen (but not peptide) bonds	0.35	1.18	0.2154	0.6597
GO:0140098	catalytic activity, acting on RNA	0.32	1.29	0.0533	0.5589
GO:0016491	oxidoreductase activity	0.32	1.46	0.0000	0.3762
GO:0044389	ubiquitin-like protein ligase binding	0.29	1.16	0.1995	0.6883
GO:0008134	transcription factor binding	0.27	1.20	0.1137	0.6839
GO:0003723	RNA binding	0.26	1.23	0.0326	0.6509
GO:0003682	chromatin binding	-0.34	-1.35	0.0361	0.3908
GO:0005198	structural molecule activity	-0.35	-1.42	0.0176	0.3057
GO:0030545	receptor regulator activity	-0.41	-1.57	0.0000	0.1825
GO:0005126	cytokine receptor binding	-0.41	-1.49	0.0181	0.3001
GO:0019955	cytokine binding	-0.41	-1.37	0.0660	0.3777
GO:0030246	carbohydrate binding	-0.42	-1.42	0.0470	0.3384
GO:0001664	G protein-coupled receptor binding	-0.43	-1.45	0.0376	0.3193

GO:0017171	serine hydrolase activity	-0.50	-1.64	0.0051	0.2073
GO:0019838	growth factor binding	-0.51	-1.61	0.0173	0.1785
GO:0016765	transferase activity. transferring alkyl or aryl (other than methyl) groups	-0.68	-1.82	0.0018	0.0548
**R_vs_C_KEGG**					
geneSet	description	ES	NES	pValue	FDR
ssc00190	Oxidative phosphorylation	0.60	2.35	0.0000	0.0000
ssc05012	Parkinson disease	0.57	2.29	0.0000	0.0000
ssc00620	Pyruvate metabolism	0.64	2.07	0.0000	0.0007
ssc05010	Alzheimer disease	0.46	1.92	0.0000	0.0099
ssc04623	Cytosolic DNA-sensing pathway	0.54	1.90	0.0000	0.0114
ssc01200	Carbon metabolism	0.47	1.93	0.0000	0.0118
ssc03050	Proteasome	0.60	1.85	0.0000	0.0180
ssc00020	Citrate cycle (TCA cycle)	0.56	1.85	0.0047	0.0199
ssc04137	Mitophagy	0.49	1.76	0.0000	0.0417
ssc04974	Protein digestion and absorption	-0.55	-1.90	0.0000	0.0441
ssc04714	Thermogenesis	0.39	1.72	0.0000	0.0604
ssc04512	ECM-receptor interaction	-0.49	-1.72	0.0016	0.2745
ssc05146	Amoebiasis	-0.46	-1.63	0.0032	0.2801
ssc04977	Vitamin digestion and absorption	-0.62	-1.65	0.0223	0.2819
ssc05150	Staphylococcus aureus infection	-0.56	-1.60	0.0162	0.3124
ssc04610	Complement and coagulation cascades	-0.48	-1.66	0.0048	0.3688
ssc04933	AGE-RAGE signaling pathway in diabetic complications	-0.42	-1.52	0.0130	0.3694

ssc00603	Glycosphingolipid biosynthesis	-0.73	-1.53	0.0311	0.3826
ssc03030	DNA replication	-0.53	-1.52	0.0438	0.3927
ssc05206	MicroRNAs in cancer	-0.36	-1.41	0.0214	0.4026
**R_vs_C_WIKI**					
geneSet	description	ES	NES	pValue	FDR
WP357	Fatty Acid Biosynthesis	0.71	2.03	0.0000	0.0057
WP78	TCA Cycle (aka Krebs or citric acid cycle)	0.69	1.88	0.0021	0.0363
WP3413	NOTCH1 regulation of human endothelial cell calcification	0.66	1.76	0.0092	0.1171
WP4290	Metabolic reprogramming in colon cancer	0.65	2.21	0.0000	0.0000
WP623	Oxidative phosphorylation	0.65	2.18	0.0000	0.0004
WP111	Electron Transport Chain (OXPHOS system in mitochondria)	0.59	2.26	0.0000	0.0000
WP3965	Lipid Metabolism Pathway	0.56	1.71	0.0069	0.1365
WP3942	PPAR signaling pathway	0.53	1.89	0.0000	0.0381
WP1772	Apoptosis Modulation and Signaling	0.47	1.72	0.0026	0.1377
WP231	TNF alpha Signaling Pathway	0.45	1.76	0.0029	0.1114
WP615	Senescence and Autophagy in Cancer	-0.48	-1.70	0.0049	0.1110
WP3945	TYROBP Causal Network	-0.56	-1.79	0.0035	0.0590
WP2806	Human Complement System	-0.59	-2.06	0.0000	0.0045
WP2361	Gastric Cancer Network 1	-0.61	-1.75	0.0018	0.0730
WP453	Inflammatory Response Pathway	-0.65	-1.84	0.0000	0.0368
WP2911	miRNA targets in ECM and membrane receptors	-0.66	-1.85	0.0035	0.0377
WP47	Hedgehog Signaling Pathway	-0.69	-1.78	0.0036	0.0643

WP545	Complement Activation	-0.72	-1.86	0.0017	0.0437
WP3877	Simplified Depiction of MYD88 Distinct Input-Output Pathway	-0.77	-1.92	0.0017	0.0247
WP3876	BMP2-WNT4-FOXO1 Pathway in Human Primary Endometrial Stromal Cell Differentiation	-0.81	-1.70	0.0091	0.1232
**R_vs_C_BP**					
geneSet	description	ES	NES	pValue	FDR
GO:0051082	unfolded protein binding	0.54	1.72	0.0072	0.1560
GO:0031072	heat shock protein binding	0.50	1.47	0.0529	0.5172
GO:0051117	ATPase binding	0.49	1.39	0.0825	0.4622
GO:0016874	ligase activity	0.37	1.33	0.0754	0.5332
GO:0016810	hydrolase activity, acting on carbon-nitrogen (but not peptide) bonds	0.35	1.21	0.1766	0.6621
GO:0140098	catalytic activity, acting on RNA	0.32	1.28	0.0641	0.5732
GO:0016491	oxidoreductase activity	0.32	1.45	0.0035	0.3994
GO:0044389	ubiquitin-like protein ligase binding	0.29	1.18	0.1474	0.6727
GO:0008134	transcription factor binding	0.27	1.18	0.1199	0.6171
GO:0003723	RNA binding	0.26	1.22	0.0471	0.6902
GO:0003682	chromatin binding	-0.34	-1.35	0.0434	0.4104
GO:0005198	structural molecule activity	-0.35	-1.43	0.0167	0.2926
GO:0030545	receptor regulator activity	-0.41	-1.57	0.0030	0.2049
GO:0005126	cytokine receptor binding	-0.41	-1.49	0.0187	0.3010
GO:0019955	cytokine binding	-0.41	-1.36	0.0779	0.4057
GO:0030246	carbohydrate binding	-0.42	-1.44	0.0306	0.3117
GO:0001664	G protein-coupled receptor binding	-0.43	-1.45	0.0551	0.3378

GO:0017171	serine hydrolase activity	-0.50	-1.63	0.0050	0.2185
GO:0019838	growth factor binding	-0.51	-1.61	0.0174	0.1764
GO:0016765	transferase activity, transferring alkyl or aryl (other than methyl) groups	-0.68	-1.82	0.0000	0.0390
**R_vs_C_CC**					
geneSet	description	ES	NES	pValue	FDR
GO:0070469	respiratory chain	0.67	2.21	0.0000	0.0000
GO:0032993	protein-DNA complex	0.37	1.17	0.2049	0.2925
GO:0120114	Sm-like protein family complex	0.36	1.18	0.2076	0.3107
GO:0043209	myelin sheath	0.36	1.33	0.0791	0.1972
GO:0042579	microbody	0.36	1.14	0.2602	0.3159
GO:0044429	mitochondrial part	0.33	1.52	0.0037	0.1664
GO:0031975	envelope	0.32	1.48	0.0000	0.1494
GO:0099568	cytoplasmic region	0.30	1.21	0.1216	0.3169
GO:1902494	catalytic complex	0.29	1.45	0.0000	0.1359
GO:0098796	membrane protein complex	0.29	1.39	0.0000	0.1655
GO:0005694	chromosome	-0.29	-1.20	0.1010	0.5863
GO:0005615	extracellular space	-0.29	-1.27	0.0432	0.6209
GO:0009986	cell surface	-0.30	-1.22	0.0892	0.6174
GO:0099080	supramolecular complex	-0.30	-1.23	0.0994	0.6772
GO:0043235	receptor complex	-0.33	-1.22	0.1329	0.5883
GO:0098552	side of membrane	-0.37	-1.46	0.0089	0.2212
GO:0034399	nuclear periphery	-0.45	-1.36	0.0819	0.3708

GO:0031012	extracellular matrix	-0.46	-1.70	0.0000	0.0381
GO:0042383	sarcolemma	-0.63	-1.78	0.0068	0.0199
GO:0005581	collagen trimer	-0.76	-1.90	0.0000	0.0115
**R_vs_C_MF**					

geneSet	description	ES	NES	pValue	FDR
GO:0051082	unfolded protein binding	0.54	1.80	0.0000	0.0765
GO:0031072	heat shock protein binding	0.50	1.54	0.0165	0.3281
GO:0051117	ATPase binding	0.49	1.42	0.0686	0.3714
GO:0016874	ligase activity	0.37	1.34	0.0781	0.4791
GO:0016810	hydrolase activity. acting on carbon-nitrogen (but not peptide) bonds	0.35	1.18	0.2154	0.6597
GO:0140098	catalytic activity, acting on RNA	0.32	1.29	0.0533	0.5589
GO:0016491	oxidoreductase activity	0.32	1.46	0.0000	0.3762
GO:0044389	ubiquitin-like protein ligase binding	0.29	1.16	0.1995	0.6883
GO:0008134	transcription factor binding	0.27	1.20	0.1137	0.6839
GO:0003723	RNA binding	0.26	1.23	0.0326	0.6509
GO:0003682	chromatin binding	-0.34	-1.35	0.0361	0.3908
GO:0005198	structural molecule activity	-0.35	-1.42	0.0176	0.3057
GO:0030545	receptor regulator activity	-0.41	-1.57	0.0000	0.1825
GO:0005126	cytokine receptor binding	-0.41	-1.49	0.0181	0.3001
GO:0019955	cytokine binding	-0.41	-1.37	0.0660	0.3777
GO:0030246	carbohydrate binding	-0.42	-1.42	0.0470	0.3384
GO:0001664	G protein-coupled receptor binding	-0.43	-1.45	0.0376	0.3193

GO:0017171	serine hydrolase activity	-0.50	-1.64	0.0051	0.2073
GO:0019838	growth factor binding	-0.51	-1.61	0.0173	0.1785
GO:0016765	transferase activity. transferring alkyl or aryl (other than methyl) groups	-0.68	-1.82	0.0018	0.0548
**B_vs_C_KEGG**					
geneSet	description	ES	NES	pValue	FDR
ssc05332	Graft-versus-host disease	0.77	1.74	0.0076	0.0446
ssc05310	Asthma	0.77	1.92	0.0000	0.0080
ssc05330	Allograft rejection	0.76	1.88	0.0055	0.0135
ssc04672	Intestinal immune network for IgA production	0.73	2.04	0.0000	0.0051
ssc04612	Antigen processing and presentation	0.65	1.98	0.0000	0.0033
ssc05340	Primary immunodeficiency	0.64	1.81	0.0038	0.0267
ssc05416	Viral myocarditis	0.63	1.97	0.0000	0.0036
ssc05140	Leishmaniasis	0.60	2.02	0.0000	0.0015
ssc04650	Natural killer cell mediated cytotoxicity	0.58	2.04	0.0000	0.0025
ssc04670	Leukocyte transendothelial migration	0.55	2.02	0.0000	0.0020
ssc05231	Choline metabolism in cancer	-0.31	-1.21	0.1371	0.6651
ssc04714	Thermogenesis	-0.33	-1.45	0.0125	0.5613
ssc04350	TGF-beta signaling pathway	-0.38	-1.45	0.0134	0.6157
ssc03010	Ribosome	-0.40	-1.51	0.0134	0.6125
ssc00190	Oxidative phosphorylation	-0.47	-1.82	0.0000	0.1819
ssc04260	Cardiac muscle contraction	-0.48	-1.52	0.0358	0.6582
ssc04960	Aldosterone-regulated sodium reabsorption	-0.51	-1.48	0.0434	0.6247

ssc00062	Fatty acid elongation	-0.62	-1.65	0.0130	0.2697
ssc04977	Vitamin digestion and absorption	-0.63	-1.73	0.0106	0.2528
ssc00603	Glycosphingolipid biosynthesis	-0.76	-1.67	0.0210	0.3107
**B_vs_C_WIKI**					
geneSet	description	ES	NES	pValue	FDR
WP530	Cytokines and Inflammatory Response	0.75	1.90	0.0000	0.0373
WP3937	Microglia Pathogen Phagocytosis Pathway	0.66	2.03	0.0018	0.0102
WP619	Type II interferon signaling (IFNG)	0.64	1.76	0.0076	0.1218
WP3945	TYROBP Causal Network	0.63	2.12	0.0000	0.0080
WP24	Peptide GPCRs	0.61	1.96	0.0000	0.0239
WP430	Statin Pathway	0.59	1.67	0.0169	0.2051
WP4136	Fibrin Complement Receptor 3 Signaling Pathway	0.57	1.78	0.0037	0.1115
WP4298	Viral Acute Myocarditis	0.55	1.90	0.0000	0.0310
WP3624	Lung fibrosis	0.52	1.72	0.0055	0.1680
WP3863	T-Cell antigen Receptor (TCR) pathway during Staphylococcus aureus infection	0.49	1.67	0.0019	0.2142
WP4222	Phosphodiesterases in neuronal function	-0.49	-1.68	0.0064	0.2748
WP111	Electron Transport Chain (OXPHOS system in mitochondria)	-0.50	-1.91	0.0000	0.0278
WP4150	Wnt Signaling in Kidney Disease	-0.53	-1.61	0.0176	0.3747
WP143	Fatty Acid Beta Oxidation	-0.56	-1.68	0.0064	0.3241
WP4324	Mitochondrial complex I assembly model OXPHOS system	-0.59	-1.98	0.0000	0.0147
WP3640	Imatinib and Chronic Myeloid Leukemia	-0.59	-1.60	0.0227	0.3473
WP623	Oxidative phosphorylation	-0.60	-2.00	0.0000	0.0253

WP2881	Estrogen Receptor Pathway	-0.63	-1.58	0.0306	0.3596
WP3876	BMP2-WNT4-FOXO1 Pathway in Human Primary Endometrial Stromal Cell Differentiation	-0.72	-1.55	0.0286	0.3892
WP3930	EDA Signalling in Hair Follicle Development	-0.72	-1.65	0.0085	0.2927
**B_vs_C_BP**					
geneSet	description	ES	NES	pValue	FDR
GO:2001057	reactive nitrogen species metabolic process	0.64	1.64	0.0161	0.1203
GO:0042107	cytokine metabolic process	0.59	1.73	0.0097	0.1154
GO:0002252	immune effector process	0.43	1.77	0.0000	0.1357
GO:0001816	cytokine production	0.41	1.73	0.0000	0.1429
GO:0009607	response to biotic stimulus	0.39	1.66	0.0018	0.1310
GO:0006952	defense response	0.38	1.70	0.0000	0.1250
GO:0080134	regulation of response to stress	0.37	1.69	0.0000	0.1114
GO:0030162	regulation of proteolysis	0.37	1.56	0.0017	0.1828
GO:0001775	cell activation	0.37	1.60	0.0000	0.1645
GO:0006955	immune response	0.37	1.65	0.0000	0.1281
GO:1990845	adaptive thermogenesis	-0.19	-0.63	0.9594	0.9853
GO:0006979	response to oxidative stress	-0.28	-1.14	0.2023	0.9521
GO:0072521	purine-containing compound metabolic process	-0.29	-1.21	0.1079	0.9893
GO:0043543	protein acylation	-0.31	-1.17	0.2000	0.8848
GO:0006836	neurotransmitter transport	-0.31	-1.14	0.2014	0.9168
GO:0007033	vacuole organization	-0.33	-1.13	0.2739	0.8978
GO:0031929	TOR signaling	-0.36	-1.17	0.2266	0.9866

GO:0006081	cellular aldehyde metabolic process	-0.46	-1.17	0.2430	0.9370
GO:0042157	lipoprotein metabolic process	-0.48	-1.51	0.0380	0.7017
GO:0140053	mitochondrial gene expression	-0.54	-1.73	0.0000	0.2001

**B_vs_C_CC**					
geneSet	description	ES	NES	pValue	FDR
GO:0098862	cluster of actin-based cell projections	0.48	1.19	0.2556	0.8289
GO:0030496	midbody	0.36	1.12	0.2865	0.9023
GO:0019898	extrinsic component of membrane	0.35	1.24	0.1356	0.9622
GO:0005615	extracellular space	0.32	1.46	0.0016	0.6314
GO:0005694	chromosome	0.28	1.22	0.0914	0.8475
GO:0017053	transcriptional repressor complex	0.27	0.77	0.8171	0.9736
GO:0048475	coated membrane	0.27	0.80	0.7790	0.9759
GO:0009986	cell surface	0.26	1.12	0.2126	0.8119
GO:0005793	endoplasmic reticulum-Golgi intermediate compartment	0.25	0.67	0.9009	0.9681
GO:0005773	vacuole	0.21	0.87	0.7578	0.9686
GO:0031975	envelope	-0.25	-1.15	0.1230	0.3803
GO:0005794	Golgi apparatus	-0.26	-1.20	0.0543	0.3338
GO:0098588	bounding membrane of organelle	-0.26	-1.21	0.0440	0.3452
GO:0031984	organelle subcompartment	-0.28	-1.32	0.0052	0.3499
GO:0042175	nuclear outer membrane-endoplasmic reticulum membrane network	-0.29	-1.28	0.0290	0.3808
GO:0099080	supramolecular complex	-0.29	-1.24	0.0681	0.3957
GO:0045177	apical part of cell	-0.32	-1.21	0.1405	0.3733

GO:1990351	transporter complex	-0.35	-1.23	0.1425	0.3679
GO:0070469	respiratory chain	-0.56	-1.83	0.0043	0.0158
GO:0042383	sarcolemma	-0.68	-2.03	0.0000	0.0038
**B_vs_C_MF**					
geneSet	description	ES	NES	pValue	FDR
GO:0140097	catalytic activity. acting on DNA	0.54	1.77	0.0000	0.0947
GO:0001653	peptide receptor activity	0.51	1.72	0.0087	0.0847
GO:0070851	growth factor receptor binding	0.38	1.16	0.2583	0.5791
GO:0003707	steroid hormone receptor activity	0.38	1.14	0.2571	0.5502
GO:0051219	phosphoprotein binding	0.37	1.14	0.2584	0.5321
GO:0016798	hydrolase activity, acting on glycosyl bonds	0.37	1.15	0.2585	0.5723
GO:0003690	double-stranded DNA binding	0.26	1.15	0.1521	0.5662
GO:0001067	regulatory region nucleic acid binding	0.26	1.13	0.1672	0.5402
GO:0046983	protein dimerization activity	0.25	1.13	0.1732	0.5572
GO:0042802	identical protein binding	0.24	1.11	0.1719	0.5532
GO:0005215	transporter activity	-0.25	-1.17	0.0821	0.7660
GO:0005198	structural molecule activity	-0.26	-1.15	0.1562	0.7062
GO:0048037	cofactor binding	-0.27	-1.16	0.1512	0.7316
GO:0016746	transferase activity, transferring acyl groups	-0.29	-1.10	0.2545	0.7471
GO:0016491	oxidoreductase activity	-0.29	-1.34	0.0105	0.6228
GO:0016757	transferase activit,. transferring glycosyl groups	-0.35	-1.34	0.0694	0.5258
GO:0002020	protease binding	-0.36	-1.12	0.2911	0.7365

GO:0101005	ubiquitinyl hydrolase activity	-0.43	-1.35	0.0797	0.7979
GO:0016209	antioxidant activity	-0.52	-1.66	0.0087	0.2363
GO:0016765	transferase activity, transferring alkyl or aryl (other than methyl) groups	-0.57	-1.60	0.0282	0.1965
